# Perinatal Exposure to a High-Fat Diet Is Associated with Reduced Hepatic Sympathetic Innervation in One-Year Old Male Japanese Macaques

**DOI:** 10.1371/journal.pone.0048119

**Published:** 2012-10-30

**Authors:** Wilmon F. Grant, Lindsey E. Nicol, Stephanie R. Thorn, Kevin L. Grove, Jacob E. Friedman, Daniel L. Marks

**Affiliations:** 1 Neuroscience Graduate Program, Oregon Health & Science University, Portland, Oregon, United States of America; 2 Department of Pediatrics, Oregon Health & Science University, Portland, Oregon, United States of America; 3 Oregon National Primate Research Center, Oregon Health & Science University, Portland, Oregon, United States of America; 4 Papé Family Pediatric Research Institute, Oregon Health & Science University, Portland, Oregon, United States of America; 5 Department of Pediatrics, University of Colorado Denver, Aurora, Colorado, United States of America; CRCHUM-Montreal Diabetes Research Center, Canada

## Abstract

Our group recently demonstrated that maternal high-fat diet (HFD) consumption is associated with non-alcoholic fatty liver disease, increased apoptosis, and changes in gluconeogenic gene expression and chromatin structure in fetal nonhuman primate (NHP) liver. However, little is known about the long-term effects that a HFD has on hepatic nervous system development in offspring, a system that plays an important role in regulating hepatic metabolism. Utilizing immunohistochemistry and Real-Time PCR, we quantified sympathetic nerve fiber density, apoptosis, inflammation, and other autonomic components in the livers of fetal and one-year old Japanese macaques chronically exposed to a HFD. We found that HFD exposure *in-utero* and throughout the postnatal period (HFD/HFD), when compared to animals receiving a CTR diet for the same developmental period (CTR/CTR), is associated with a 1.7 fold decrease in periportal sympathetic innervation, a 5 fold decrease in parenchymal sympathetic innervation, and a 2.5 fold increase in hepatic apoptosis in the livers of one-year old male animals. Additionally, we observed an increase in hepatic inflammation and a decrease in a key component of the cholinergic anti-inflammatory pathway in one-year old HFD/HFD offspring. Taken together, these findings reinforce the impact that continuous exposure to a HFD has in the development of long-term hepatic pathologies in offspring and highlights a potential neuroanatomical basis for hepatic metabolic dysfunction.

## Introduction

Fetal and early postnatal development are critical periods when perturbations to the intrauterine environment can have lifelong effects on the structure and function of organs, tissues and body systems in the offspring through long lasting changes in the developmental program [1]–[3]. Given the increasing prevalence of obesity in both adults and children, programming of developing tissues from excess maternal nutrients has been implicated as a contributing etiology to the global epidemic of obesity and metabolic disease [4]–[7].

To date, the mechanisms mediating the maternal influence over metabolic disorders in offspring is unknown, but likely includes high levels of maternal hormones (insulin and leptin) and/or nutrients (fatty acid/triglycerides and glucose) as well as changes in placental function effecting blood flow and nutrient transport [8], [9]. Nonetheless, emerging data point to both gestational and early postnatal nutritional cues as being critical to the development of systems that regulate body weight, energy and glucose homeostasis in offspring.

Recent work by our group has revealed in the nonhuman primate (NHP) that chronic exposure to a maternal high-fat diet (HFD) is associated with alterations in the fetal liver that include modifications of hepatic chromatin structure, alterations in gluconeogenic and circadian gene expression, increased apoptosis, elevated triglyceride content and evidence of oxidative stress and nonalcoholic fatty liver disease (NAFLD) [10]–[13]. Changes in central serotonergic systems and alterations in the development and expression of hypothalamic neuropeptides reported in fetal and one-year old HFD NHP offspring, have also revealed an abnormal development of brain circuits critically involved in the autonomic regulation of glucose homeostasis with this model [14], [15].

Pioneering work by Shimazu et al., introduced the role that the central and the peripheral nervous systems play in the regulation of hepatic metabolism [16]–[19]. Despite more recent evidence defining the autonomic regulation of hepatic glucose production, the role that the autonomic nervous system plays in the control of hepatic metabolism as a whole, remains unclear [20]–[22]. Further, there are many details regarding the neuroanatomy of the liver itself that still remain to be addressed [23]. Significantly, there is very little data describing the autonomic innervation of the NHP liver. While similarities exist in the hepatic innervation between adult humans and the adult NHP [24], [25], the lack of more detailed knowledge in hepatic neuroanatomy or hepatic autonomic development in the NHP is a major obstacle for functional studies of liver metabolism in these animals.

The current study extends previous work by our group and examines the relationship between the HFD and the development of the sympathetic nervous system in NHP liver. In this study, we report that neuropeptide-Y (NPY) immunoreactive nerve fibers are present and localized to the portal triad in fetal macaque liver. In addition, we observed that robust sympathetic innervation is present in portal and parenchymal regions in the one-year old macaque. Importantly, we demonstrate that continuous exposure to a high-fat diet *in-utero* and through the post-natal period (HFD/HFD) is associated with increased hepatic apoptosis and decreased hepatic sympathetic innervation in parenchymal and portal regions in one-year old male macaques. We also report, that increased hepatic expression of inflammation is observed along with decreased expression of an integral component of the cholinergic anti-inflammatory pathway in the HFD/HFD juveniles. These data suggest that permanent changes in hepatic sympathetic innervation, and consequent functional effects, may be present in the HFD/HFD juvenile males. In addition, parasympathetic mechanisms shown to inhibit inflammation may also be altered in the HFD/HFD animals.

## Materials and Methods

### Ethics Statement

All animal procedures have undergone an extensive review process and were in accordance with the guidelines of Institutional Animal Care and Use Committee of the Oregon National Primate Research Center (ONPRC) and Oregon Health & Science University. Protocols involved in this study were developed to ameliorate suffering and have been approved under IACUC ID number: IS00000224 (0622 for internal purposes). The Animal Care and Use Program at the ONPRC abides by the Animal Welfare Act and Regulations (CFR 9, Ch 1, Subchapter A) enforced by the USDA, the Public Health Service Policy on Humane Care and Use of Laboratory Animals, in accordance with the *Guide for the Care and Use of Laboratory Animals* of the National Institutes of Health, and the recommendations of the Weatherall report; *The Use of Non-human Primates in Research*.

### Animals

Our model and materials and methods for fetal studies has previously been described in detail [11], [12]. Briefly, Japanese macaques matched for age (5–7 years at start) and weight (7–9 Kg) were randomly assigned to two dietary groups: 1: Control diet (CTR; 13% of calories from fat; Monkey Diet no. 5052, Lab Diet, Richmond, IN, USA) or 2: High-fat diet (HFD; 35.2% of calories from fat; Custom Diet 5A1F, Test Diet, Richmond, IN, USA). The HFD also included calorically dense treats made with peanut butter. Both diets are sufficient in vitamin, mineral, and protein content for normal growth. Prior to this study, all animals were maintained on standard monkey chow in large outdoor enclosures and were naive to any experimental protocols.

The animals were group housed and had *ad libitum* access to food and water. Each maternal group was housed with two males and the females were checked each successive year for pregnancies during the breeding season by ultrasound, which allows an estimate of gestational age ±5 days. Twice a year the animals underwent IV glucose tolerance tests (IVGTT), once during the late summer (nonpregnant state) and once during the early 3rd trimester of pregnancy. All of the above procedures were done under ketamine sedation (5–10 mg/kg).

For fetal studies, ONPRC veterinarians terminated singleton pregnancies from dams by cesarean section at gestational day 130 (G130), as determined by ultrasound. Normal full-term pregnancies for Japanese macaques is 175 days, thus G130 is in the early 3^rd^ trimester. Pregnant dams were fasted overnight for approximately 16 hours prior to surgical procedure. Females were initially sedated with ketamine hydrochloride (100 mg/ml) at a dose of 10–15 mg/kg. Once animals were sedated they were delivered to the surgical area and placed on isoflurane gas; induced at 3%, then maintained at 1.0–1.5%. Cesarean sections were performed by trained ONPRC veterinarians and their staff. After cesarean section, fetuses were deeply anesthetized with sodium pentobarbital (>30 mg/kg i.v.) and exsanguinated. The liver was removed, weighed and the right lobe was stored for the subsequent RNA extractions and histological analyses used in this study. Pre and post-operative care was maintained by ONPRC veterinary staff. All surgical procedures used in this study, were performed each scheduled day in an identical manner, following a consistent routine in both technique and timing.

For our fetal studies, we are reporting differences in HFD fetuses whose mothers were exposed to the maternal diet for at least four consecutive years. In the fifth year of our studies, a diet-reversal protocol (REV) was initiated to assess dietary impact independent of maternal obesity. This protocol entailed switching a subgroup of adult females that had been exposed to a high-fat diet for four consecutive years, to a control diet 1–3 months before becoming pregnant and throughout the pregnancy. The CTR cohort contained 6–8 animals (2–5 females, 3–4 males), the HFD cohort contained 8 animals (4 females, 4 males) and the REV cohort contained 7 animals (5 females, 2 males).

**Table 1:** Inflammatory marker mRNA expression in juvenile liver.^1^

TARGET	DIET GROUP				*p*-value^2^
	CTR/CTR		HFD/HFD		
	Mean	SEM	Mean	SEM	
C-Reactive protein	2.95	0.86	6.50	1.95	0.13
Interferon-γ	1.03	0.15	0.93	0.13	0.73
Interleukin-1β	0.97	0.14	1.23	0.17	0.35
Interleukin-10	1.38	0.22	1.09	0.15	0.33
I-TAC (CXCL11)	0.60	0.09	0.77	0.15	0.64
Lymphotoxin-α	**1.02**	0.11	**1.47**	0.16	**0.02**
MCP-1 (CCL2)	0.70	0.12	0.65	0.04	0.73
Tumor NecrosisFactor-α	0.93	0.13	1.40	0.21	0.15

1 All values are means ± SEMs and are expressed as relative fold to CTR calibrator sample. n = 7−10 for CTR, n = 8−10 for HFD.

2 Overall significance as determined by Kruskal-Wallis rank sum test.

For our juvenile studies, full-term offspring were maintained with their birth mothers on the same diet as consumed during pregnancy until weaning. Between 7 and 8 months, the offspring were weaned to create diet cohorts with the first dietary designation indicating the maternal diet *in-utero* and before weaning, and the second designation indicating the offspring’s diet after weaning. The two juvenile diet groups examined in this study were CTR/CTR and HFD/HFD. The CTR/CTR cohort contained 7 males and 5 females and the HFD/HFD cohort contained 6 males and 4 females.

The mean age of the juvenile offspring at time of necropsy was 12.9 months with a 95% confidence interval of between 12.7–13.1 months. Necropsy was carried out by an *a-priori* protocol by the veterinary staff at ONPRC as previously described [11], [12], [15]. The liver was removed, weighed and the right lobe was stored for the subsequent RNA extractions and histological analyses used in this study.

**Figure 1:**
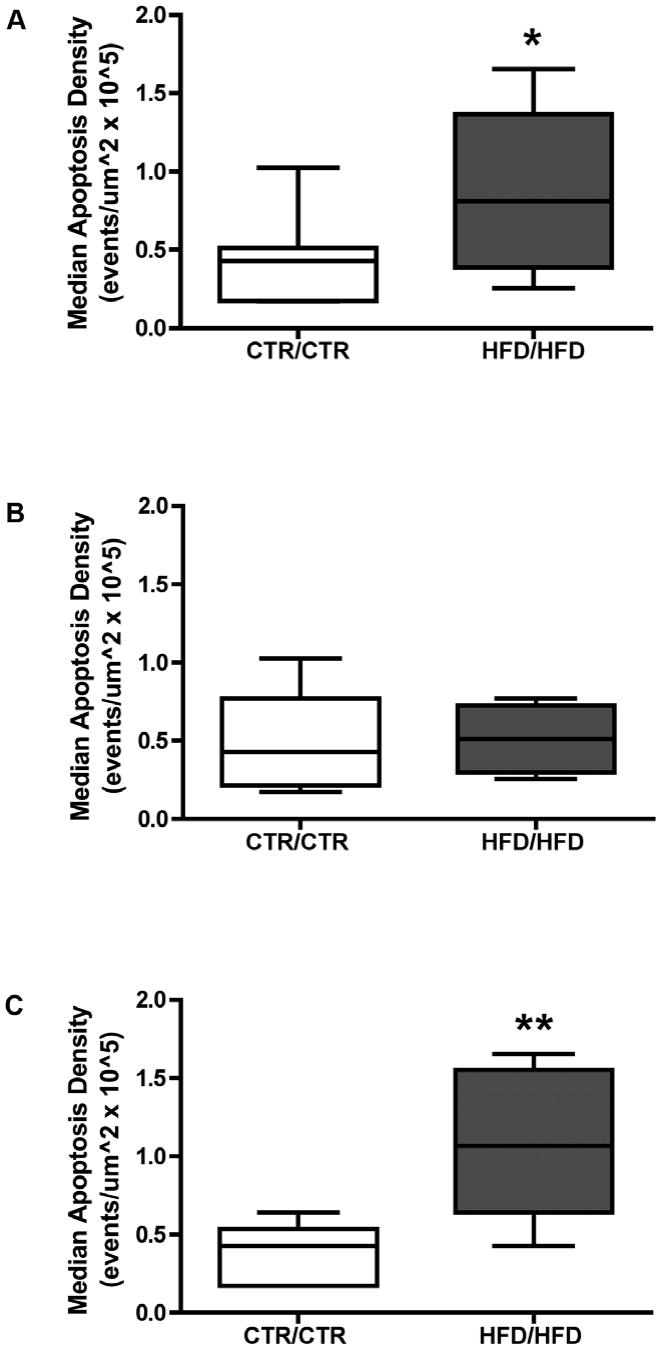
Quantification of hepatic apoptosis between CTR/CTR and HFD/HFD juvenile macaques as determined by TUNEL staining. (A) Quantification of hepatic apoptosis between entire cohort of juvenile macaques. (B) Comparison of apoptosis in female juvenile macaque liver between CTR/CTR and HFD/HFD diet groups. (C) Quantification of apoptosis in male juvenile macaque liver between CTR/CTR and HFD/HFD diet groups. CTR/CTR n = 11, 6 males 5 females; HFD/HFD n = 10, 6 males, 4 females. (* = *p*<0.05, ** = *p*<0.01).

### Real-Time PCR

Reactions were run on an Applied Biosystems 7300 as relative quantification plates using macaque specific primers as previously described [11]. Additional primers were designed to quantify autonomic targets of interest (Definition of abbreviations: F, forward primer; R, reverse primer):

Alpha 1A Adrenergic Receptor (ADRA1A; F: CGACACCTGCACTCAGTCACA,

R: CCTCGAAGATGGCGGAGAA, Genbank Accesion No.: NW_001122890),

Alpha 1B Adrenergic Receptor (ADRA1B; F: CAGCTAAGACGTTGGGCATTG,

R: GGCTTCAGGGTGGAGAACAA, Genbank Accesion No.: NW_001120992),

Alpha 2A Adrenergic Receptor (ADRA2A; F: CTGGTGGCCACGCTTGTC,

R: CGTCGAGCGCCAGGTAGAT, Genbank Accesion No.: NW_001124223),

Type-1 Cannabinoid Receptor (CB1R; F: ACGCTTTCCGGAGCATGTT,

R: GCGTTGTTTGCGTGTTTGTG, Genbank Accesion No.: NM_001032825),

Type-2 Cannabinoid Receptor (CB2R; F: GGGCATGTTCTCTGGAAAGC,

R: ACCTCACGTCCAGCCTCATT, Genbank Accesion No.: NW_001111036),

Cholinergic Receptor, Nicotinic, Alpha 7 (CHRNA7; F: TGGTGGTGACGGTGATCGT,

R: CACGCGCACCAGTTCAGA, Genbank Accesion No.: NM_001032883),

Neuropeptide-Y Y1 Receptor (NPY1R; F: ATTTCCGGTCTCGGGATGAT,

R: AATGCGACTGGGCTTGCTT, Genbank Accesion No.: NM_001032866),

Glucose Transporter 2 (GLUT2; F: GACCACGTCCTGCTGCTTTAG,

R: GGTCCACAGAAGTCCGCAAT, Genbank Accesion No.: NW_001112558).

Real-Time PCR reaction products were run on a 2% agarose gel to verify amplicon singularity and size. Bands of the expected size were excised, gel purified (Qiaquick gel extraction kit, Qiagen #28706) and sequenced. Target specificity was confirmed by BLAST and comparing amplicon sequence with the NCBI macaque database.

**Table 2:** Peripheral Nervous System mRNA expression in Fetal Macaque Liver.^1^

Target	Common Name (Type)	DIET GROUP						*p*-value^2^
		CTR		HFD		REV		
		Mean	SEM	Mean	SEM	Mean	SEM	
ADRA1A	adrenergic, α1A receptor	0.91	0.06	0.93	0.11	0.85	0.12	0.69
ADRA1B	adrenergic, α1B receptor	0.89	0.05	1.00	0.09	1.08	0.10	0.28
ADRA2A	adrenergic, α2A receptor	0.67	0.07	0.63	0.07	0.79	0.07	0.33
CB1R	cannabinoid 1 receptor	0.31	0.10	0.47	0.14	0.39	0.08	0.30
CB2R	cannabinoid 2 receptor	0.73	0.11	0.85	0.18	1.18	0.18	0.19
CHRNA7	cholinergic receptor, nicotinic, α7	0.93	0.09	1.02	0.10	0.81	0.06	0.24
NPYY1R	neuropeptide-Y Y1 receptor	0.91	0.07	**0.95** c	0.06	**0.66** b	0.04	**0.01**
GLUT2	glucose transporter 2	1.33	0.10	1.36	0.11	1.38	0.03	0.82

1 All values are means ± SEMs and are expressed as relative fold compared to CTR. n = 7 for CTR, n = 8 for HFD, n = 7 for REV.

2 Overall significance as determined by Kruskal-Wallis rank sum test.

a Significantly different from CTR, p<.0167, Bonferroni adjusted α.

b Significantly different from HFD, p<.0167, Bonferroni adjusted α.

c Significantly different from REV, p<.0167, Bonferroni adjusted α.

### Immunohistochemistry

NPY immunoreactivity was detected with a sheep polyclonal NPY antibody (Cat. #AB1583, Lot # JC1676120, Millipore, Temecula, CA, 1∶4000). Tyrosine hydroxylase immunoreactivity was detected with mouse monoclonal ascites (Cat. # MAB318 - clone LNC1, Millipore, Temecula, CA, 1∶200). Both antibodies have been previously characterized for specificity in the macaque and antibody control reactions were also employed in this study [26], [27].

**Table 3:** Peripheral Nervous System mRNA expression in Juvenile Macaque Liver.^1^

Target	Common Name (Type)	DIET GROUP				*p*-value^2^
		CTR/CTR		HFD/HFD		
		Mean	SEM	Mean	SEM	
ADRA1A	adrenergic, alpha-1A-, receptor	1.34	0.08	1.29	0.09	0.56
ADRA1B	adrenergic, alpha-1B-, receptor	0.86	0.08	0.82	0.07	0.73
ADRA2A	adrenergic, alpha-2A-, receptor	0.88	0.13	0.97	0.08	0.41
CB1R	cannabinoid 1 receptor	0		0		N/A
CB2R	cannabinoid 2 receptor	1.26	0.13	1.69	0.23	0.11
CHRNA7	cholinergic receptor, nicotinic, alpha 7	**0.86**	0.06	**0.51**	0.03	**0.001**
NPYY1R	neuropeptide-Y Y1 receptor	0.72	0.06	0.83	0.08	0.30
GLUT2	glucose transporter 2	0.95	0.04	0.81	0.05	0.08

1 All values are means ± SEMs and are expressed as relative fold compared to CTR/CTR. (n = 7−10 for CTR/CTR, n = 8−10 for HFD/HFD).

2 Overall significance as determined by Kruskal-Wallis rank sum test.

Liver tissue postfixed in 10% zinc formalin was embedded in paraffin and sectioned at 16 µm. Sections were deparaffinized with xylene and rehydrated in a descending alcohol series (100%, 70%, 50%). Heat induced epitope retrieval (HIER) was performed with a citrate based antigen unmasking solution (100X, Vector Laboratories, cat. # H-3300) for approximately 15 minutes in a commercial autoclave. Following HIER, sections were washed in 1X PBST (1X PBS with 0.05% Tween 20) and blocked for 1 hour at room temperature in 5% normal serum (NPY; donkey, TH; goat). Primary antibodies were incubated for 48 hrs at 4°C in a humidified chamber. Sections were then washed in 1X PBST and the appropriate secondary antibody (Alexa-fluor donkey anti-sheep 555, Alexa-fluor goat anti-mouse 488 or 555, Molecular Probes, Invitrogen), diluted 1∶500 in PBST was applied for 1 hr at room temperature. For doubled labeled immunohistochemistry, a cocktail of each antibody was used. Sections were washed in 1X PBST and coverslips were applied using elvanol mounting medium.

**Table 4:** Summary of Significant Results in Fetal and Juvenile Liver.

	FETAL						JUVENILE			
	CTR		HFD		REV		CTR/CTR		HFD/HFD	
PARAMETER	Male	Female	Male	Female	Male	Female	Male	Female	Male	Female
Apoptosis	Previously Evaluated^3^								Increased	
Steatosis	Previously Evaluated^4^						No Change			
LTA	Previously Evaluated^3^								Increased	
CRP	Previously Evaluated^3^									Increased
NPYY1R					Decreased^5^		No Change			
CB1R	Expressed/No Change						Not Expressed			
CHRNA7	No Change								Decreased	
NPY Nerves^1^	Expressed						Expressed			
NPY Nerves^2^	Not Expressed						Expressed			
NPY Density^1^	No Change									
NPY Density^2^	Not Applicable									
TH Nerves^1^	Expressed						Expressed			
TH Nerves^2^	Not Expressed						Expressed			
TH Density^1^									Decreased	
TH Density^2^									Decreased	
Glycogen									Decreased	
Wet Weight									No Change	

1 PERIPORTAL.

2 PARENCHYMA.

3 Grant *et al.* PLoS One. 2011 Feb 25;6(2): e17261.

4 McCurdy *et al.* J Clin Invest. 2009 Feb;119(2): 323–35.

5 NPYY1R Significantly Decreased vs. HFD.

### Microscopy

Liver sections were de-identified and imaged with a C-APO 40X 1.2 W Corr M27 objective on an LSM710 laser-scanning confocal microscope using Zen software (Carl Zeiss, Thornwood, NY). To minimize bias between samples, we selected portal and parenchymal regions according to specific criteria. Utilizing epifluorescence from the GFP channel, which contained no observable signal of nerve staining, we visually identified portal triads that: 1) contained a distinct portal vein, hepatic artery and bile canniculi and 2) were of a similar size that would fit cleanly into our field of view. For parenchymal regions, we chose regions that had no discernable structural elements other than sinusoids and that were located roughly midway within the hepatic lobule.

**Figure 2:**
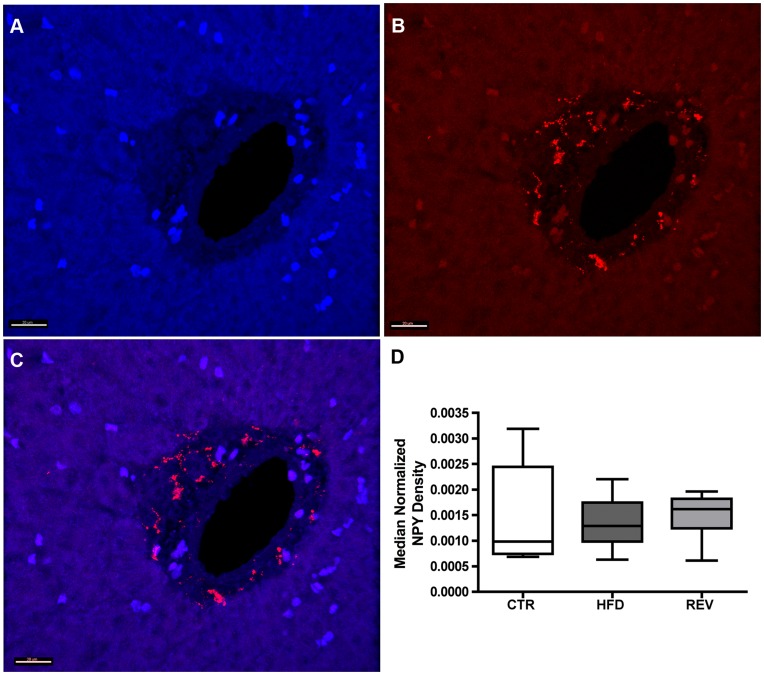
Visualization and quantification of the median density of portal NPY nerve fibers between maternal diet groups in the fetal macaque liver. (A) Tissue autofluorescence of a representative fetal portal region. (B) NPY immunofluorescence in the same portal region. (C) Overlay of NPY immunoreactivity with portal region. The volume of NPY immunoreactive fibers in each portal region (B) was quantified and normalized to the volume of hepatic tissue in each image (A). (D) No differences were observed in the density of NPY peptidergic innervation in fetal liver between maternal diet groups. Scale bar = 20 µm. CTR; n = 6, HFD; n = 8, REV; n = 7.

Image acquisition of nerve structures was performed with a 561 nm laser and acquisition parameters were optimized to maximize the signal-to noise ratio of the 555 fluorophore without saturating individual pixel signal intensity. *Z*- stacks, with a step-size of 1µm/section, were acquired for each target region using as many optical sections as necessary to image through the entire thickness of each liver section. For both fetal and juvenile studies, we acquired at least 8–10 fields for each region (portal triad, parenchyma) conforming to our selection criteria for each animal. One liver section from each animal was examined (Fetal study: 6 CTR, 8 HFD, 7 REV; Juvenile study: 12 CTR/CTR, 9 HFD/HFD). All images for the fetal and juvenile studies were acquired in an identical manner.

**Figure 3:**
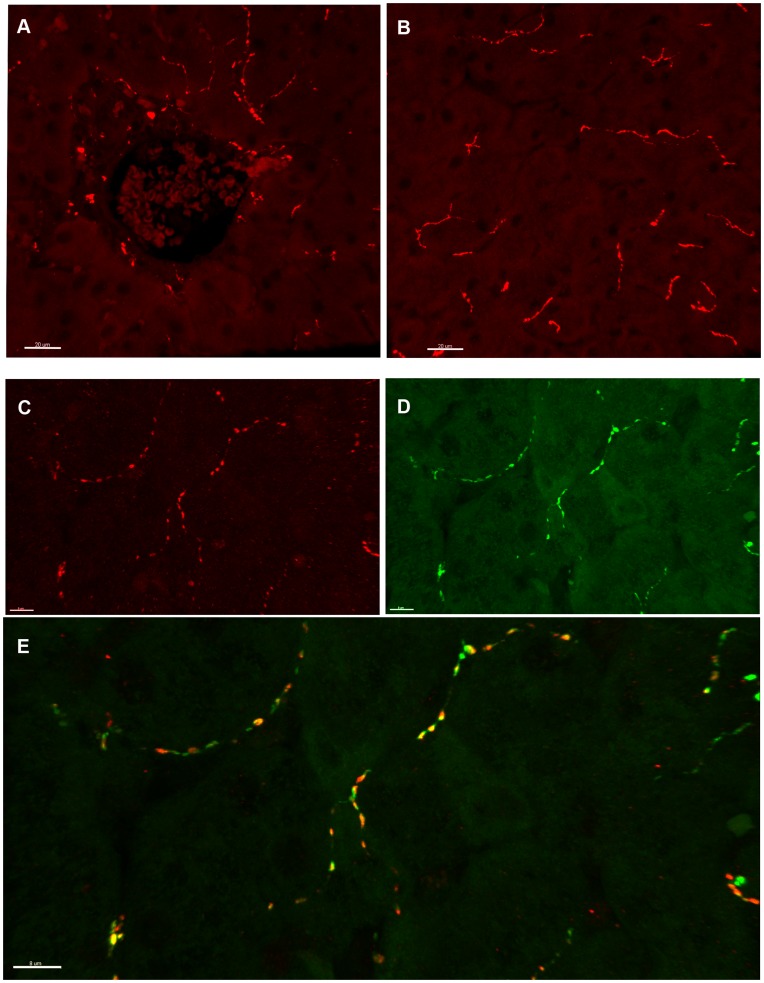
Hepatic sympathetic innervation in the one-year old juvenile macaque. (A-B) Representative image of TH immunoreactive nerve fibers in the portal region (A) and parenchyma (B) in juvenile liver. Scale bar = 20 µm. (C-E) Colocalization of NPY and TH immunoreactivity in the hepatic parenchyma of a one-year old juvenile macaque. (C) NPY immunoreactive fibers in the hepatic parenchyma. (D) TH immunoreactive fibers in the hepatic parenchyma. (E) Overlay of C and D demonstrates that TH and NPY are sympathetic in origin and are tightly colocalized in the juvenile macaque liver. Scale bar = 8 µm.

### Quantification of Nerve Fiber Density

Fetal and juvenile sympathetic nerve fiber density was quantified in an identical and blinded manner. Sympathetic nerve fiber density was normalized to hepatic volume using Imaris 7.1 image analysis software (Bitplane, Zurich Switzerland). Background autofluorescence was utilized to calculate the volume of hepatic tissue in each image by constructing a surface object with the following parameters: Smoothing 5 µm, background 9 µm, threshold.250, number of voxels above 10.

An additional surface was constructed to determine the volume of sympathetic immunoreactivity present in each image by the following parameters: Smoothing 0.2 µm, background 0.5 µm, threshold 5, maximum intensity 35, number of voxels above 10. Each image was visually inspected for the integrity of the surface calculations and the volume statistics were exported into Excel. The nerve fiber volume was then normalized to total hepatic volume for each image.

Data is expressed as the median normalized nerve density for each respective diet group. All graphs were made with Prism software (GraphPad Software, Inc., La Jolla, CA.). Representative images were created using Imaris 7.1 as maximum intensity projections and linear adjustments to brightness and contrast were made using Adobe Photoshop CS (Adobe Inc., Los Altos, CA.).

**Figure 4:**
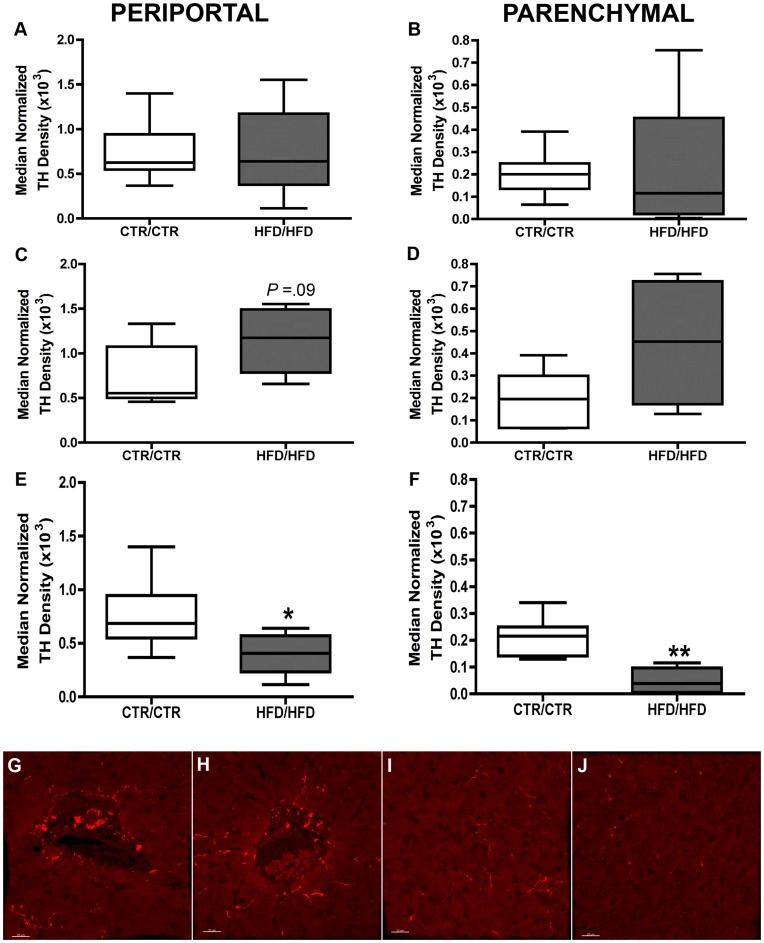
Quantification of the density of TH nerve fibers between CTR/CTR and HFD/HFD diet groups in the juvenile macaque liver. (A,C and E) Quantification of TH nerve fibers in the periportal region. (B,D and F) Quantification of TH nerve fibers in the hepatic parenchyma. TH immunofluorescence was acquired in each region by laser scanning confocal microscopy. The volume of TH immunoreactive fibers was normalized to the volume of hepatic tissue in each image. Data are expressed as the median normalized density for each juvenile diet group. (A-B) No differences were observed in the density of sympathetic innervation in juvenile liver between diet groups. CTR/CTR; n = 12, HFD/HFD; n = 9. (C-D) Quantification of the density of TH nerve fibers between CTR/CTR and HFD/HFD diet groups in the female juvenile macaque liver. A nonsignificant trend for higher sympathetic innervation was observed in the female juvenile liver between diet groups. CTR/CTR; n = 5, HFD/HFD; n = 4. (E-F) Quantification of the density of TH nerve fibers between CTR/CTR and HFD/HFD diet groups in the male juvenile macaque liver. Significantly reduced sympathetic innervation was observed between diet groups in both portal (E) and parenchymal regions (F) in the male juvenile liver. CTR/CTR; n = 7, HFD/HFD; n = 5. (* = *p*<0.05, ** = *p*<0.01). (G-J) Representative images of TH immunoreactivity in the portal region for CTR/CTR (G) and HFD/HFD (H) males. Representative images of TH immunoreactivity in the hepatic parenchyma for CTR/CTR (I) and HFD/HFD (J) males. Scale bar = 20 µm.

**Figure 5:**
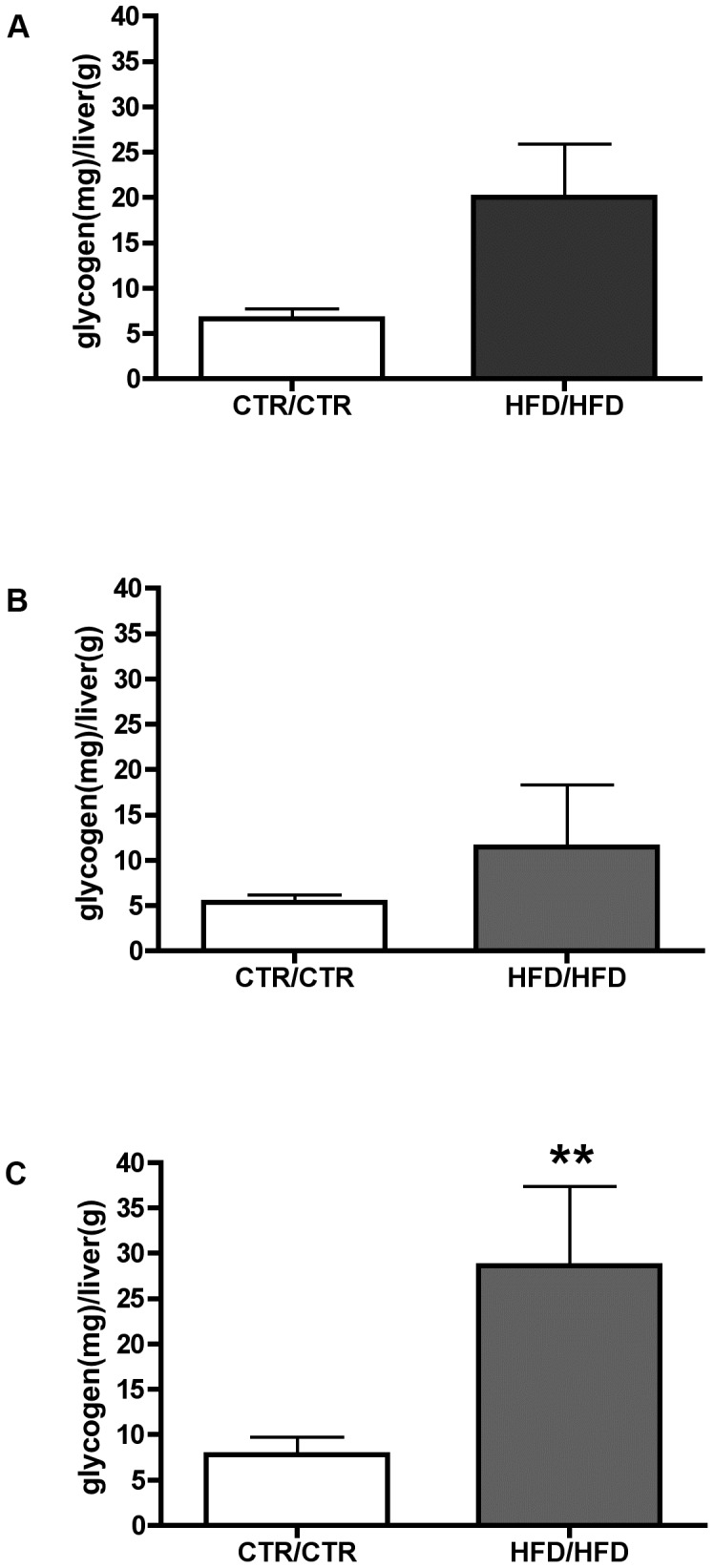
Quantification of hepatic glycogen content in the one-year old juvenile macaque. Quantification of hepatic glycogen between CTR/CTR and HFD/HFD diet groups (A). Quantification of hepatic glycogen in female juvenile livers between CTR/CTR and HFD/HFD juvenile diet groups (B). Quantification of hepatic glycogen in male juvenile livers between CTR/CTR and HFD/HFD juvenile diet groups (C). CTR/CTR n = 17, 9 males 8 females; HFD/HFD n = 14, 7 males, 7 females. (** = *p*<0.01).

### TUNEL Assay and Apoptosis Quantification

All tissue sections were treated according to the manufacturer instructions using the fluorescent staining of paraffin-embedded tissue protocol from the Apo Tag Fluorescein *In Situ* Apoptosis Detection Kit (#S7110 Millipore, Temecula, CA) without modification. Sections were counter stained with DAPI, mounted and viewed by fluorescence microscopy using a standard GFP filter at 20x with a Leica DM4000B microscope. Five random, non-overlapping digital images were obtained from each animal (11 CTR/CTR, 10 HFD/HFD) using the Leica Application Suite V3.6 and apoptotic events were counted by a blinded observer using Image J and normalized to the total surface area.

### Reverse Transcriptase-PCR

For reverse transcriptase-PCR (RT-PCR) of CHRNA7, 1.3 ug of total RNA prepared from two randomly selected juvenile livers (1 CTR/CTR, 1 HFD/HFD) was reverse transcribed in a 75 ul reaction using a Taqman Reverse Transcription kit (Applied Biosystems N808-0234) according to manufacturer’s protocol. 50 ng of cDNA was amplified using Taq platinum polymerase in a 50 µl reaction. 200 pmol of the 5′ and 3′ primers were used in a Touchdown protocol to amplify a single 500 bp band (95°C for 2 min., [(95°C for 30 s, 64°C for 30 s, 72°C for 1 min) x 20 cycles], 95°C for 30 s, [(54°C for 30 s, 72°C for 1 min) x 20 cycles], 72°C for 2 min, 4°C hold). The primers used for this reaction were: (Forward: CGCTCACCGTCTACTTCTCC, Reverse: GTAGGGCTCTTTGCAGCACT, Genbank Accesion No.: NM_001032883). PCR reaction products were run on a 1.5% agarose gel to verify amplicon singularity and size. Bands of the expected size were excised, gel purified (Qiaquick gel extraction kit, Qiagen #28706) and sequenced. Target specificity was confirmed by BLAST and comparing amplicon sequence with the NCBI macaque database.

### Oil-Red-O Staining

Fresh frozen right lobe of juvenile liver was sectioned at 10 µm, fixed in 10% zinc formalin at 4°C for 10 minutes and stained with Oil-Red-O and hematoxylin according to manufacturers instructions (American Master Tech Scientific Inc.). Sections were coded and read in a blinded manner by a board certified pathologist.

### Glycogen Assay

The right lobe of the juvenile liver was used for this procedure. Briefly, 100 mg of hepatic tissue was pulverized and digested in 2 ml of 30% KOH at 95°C for 30 min. The homogenate (150 µl) was placed on No. 1 Whatman filter paper and washed in 66% ethanol with constant stirring for 30 min. The filter paper was removed, dried, and cut into small pieces. Glycogen was converted to glucose with 31.1 U amyloglucosidase (Sigma Chemical) in 0.2 M acetate buffer (pH 4.8, 0.5% glacial acetic acid, 0.12 M sodium acetate) at 37°C for 60 min. Glucose concentration of this solution was determined in triplicate on a microplate using glucose oxidase method (Sigma) and compared with concurrently run standards of glycogen (Sigma Chemical). Results are expressed as milligrams glycogen per grams tissue (wet weight). This expanded juvenile cohort consisted of 17 CTR/CTR animals (8 females, 9 males) and 14 HFD/HFD animals (7 females, 7 males) and included all the animals used in the nerve fiber assay, TUNEL assay and Real-Time PCR experiments.

### Data Analysis

Data for all analyses were first compiled and tested for normality by Shapiro-Wilk. Data were then tested for overall significance by Kruskal-Wallis rank sum, followed by a Wilcoxon rank sum test with a Bonferroni adjusted alpha, when needed, to determine significance between multiple diet groups. Data analysis was performed with STATA (College Station, Texas) statistical software and graphs were made with Prism software (GraphPad Software, Inc., La Jolla, CA).

## Results

### Hepatic Inflammation and Apoptosis in HFD/HFD Juvenile Macaques

In previous work with this model, our group demonstrated that oxidative damage, hepatic steatosis, upregulation of phospho-JNK1 that was highly correlated with levels of fetal liver triglycerides, and increased hepatic apoptosis without evidence of hepatic inflammation was observed in HFD fetal livers [11], [12]. To extend these findings, we used Real-time PCR to evaluate hepatic apoptosis and the expression of inflammatory markers between the CTR/CTR and HFD/HFD diet groups in the juvenile liver.

We observed a significant upregulation in the expression of lymphotoxin-α (LTA, χ^2^ = 5.1, 1 d.f., *p* = 0.02) across both sexes in the HFD/HFD group when compared to CTR/CTR (**Table 1**). C-reactive protein (CRP) was not differentially expressed in the juvenile liver when the data was analyzed without regard to the sex of the animal. When the analysis was performed separately for male and female offspring, a significant increase in CRP expression was observed in the female juvenile livers (χ^2^ = 3.8, 1 d.f., *p* = 0.05, **Figure S1**). No differences were observed in the expression of interleukin-1β, interleukin-10, tumor necrosis factor-α (TNF-α), interferon-γ, monocyte chemotactic protein-1 (MCP-1, CCL2), or interferon-inducible T-cell alpha chemoattractant (I-TAC, CXCL11) between CTR/CTR and HFD/HFD diet groups.

Differences in hepatic cell death between CTR/CTR and HFD/HFD juvenile liver were evaluated by TUNEL assay. Hepatic apoptosis was significantly increased in the HFD/HFD animals when compared to CTR/CTR (χ^2^ = 5.0, 1 d.f., *p* = 0.03, **Figure 1A**) and further analysis revealed that when the sex of the animal was taken into account, no differences in apoptosis were observed between female CTR/CTR and HFD/HFD livers (χ^2^ = 0.14, 1 d.f., *p* = 0.7, **Figure 1B**). In contrast, a highly significant increase in apoptosis was observed in the male HFD/HFD liver when compared to CTR/CTR (χ^2^ = 6.4, 1 d.f., *p* = 0.01, **Figure 1C**).

A pathologist performed a blinded histological analysis of hepatic steatosis by Oil-Red-O staining. It was noted that high variability was observed in both the CTR/CTR and HFD/HFD diet groups. However, we observed no differences in steatosis between the CTR/CTR and HFD/HFD juvenile diet groups. We also found no differences when separated by sex (data not shown).

### Gene Expression of Autonomic Targets in Fetal and Juvenile Liver

To investigate the effects that a maternal HFD has on the development of the autonomic nervous system in the nonhuman primate liver, we used Real-time PCR as a screening tool to identify differential expression of autonomic targets in both fetal and one-year old juvenile livers. Within this study, we also included glucose transporter 2 (GLUT2) for its known role in vagally mediated glucose sensing in the portal vein [28].

The fetal liver had no changes in gene expression for adrenergic alpha 1A, 1B and 2A (ADRA1A, ADRA1B, ADRA2A) receptors. No differences were also observed across our three fetal dietary groups for cannabinoid 1 and 2 (CB1R, CB2R) receptors, the nicotinic cholinergic receptor alpha 7 subunit (CHRNA7) and GLUT2. However, we observed that the expression of neuropeptide-Y Y1 receptor (NPYY1R) was significantly downregulated in the diet-reversed fetuses (REV) when compared to the HFD fetuses. There was no statistical difference in NPYY1R expression between CTR and REV fetal livers (**Table 2**).

In juvenile livers, again we observed no changes in gene expression for adrenergic alpha 1A, 1B and 2A receptors. No differences were also observed for CB2R. Surprisingly, CB1R was not expressed in juvenile liver suggesting that hepatic CB1R expression is developmentally regulated in the Japanese macaque. Gene expression for NPYY1R and GLUT2 were similar between the CTR/CTR and HFD/HFD animals. In contrast, CHRNA7 expression was significantly lower in the HFD/HFD livers when compared to CTR/CTR (χ^2^ = 10.5, 1 d.f., *p* = 0.001), (**Table 3**). We observed that hepatic CHRNA7 expression was significantly decreased regardless of the sex of the HFD/HFD animal (χ^2^ = 3.9, 1 d.f., *p* = 0.0495; females, χ^2^ = 6.0, 1 d.f., *p* = 0.0143; males, **Table 4**).

While we routinely sequenced amplicons from our Real-Time PCR studies to confirm primer specificity, we also examined CHRNA7 subunit mRNA expression in the macaque liver by RT-PCR and sequence analysis. We developed primers complementary to the known macaque CHRNA7 sequence and performed RT-PCR with two randomly chosen juvenile samples. The expected 500 base pair band following electrophoresis (**Figure S2**) was gel-purified, sequenced and compared with the NCBI macaque database confirming the presence of authentic CHRNA7 subunit RNAs in the juvenile macaque liver.

### Maternal High-Fat Diet and Sympathetic Innervation in the Fetal Macaque

The distribution of Neuropeptide-Y (NPY) containing nerve fibers was evaluated in G-130 fetal liver by immunohistochemistry. NPY is a neuropeptide that has a similar distribution and co-localization with tyrosine hydroxylase (TH) immunoreactive fibers in the mammalian liver [29], [30]. Thus, it is a marker of sympathetic innervation.

Our results show that NPY immunoreactive fibers are present in fetal macaque liver. The distribution of NPY immunoreactive fibers are localized to the portal triads and very rarely observed in the hepatic parenchyma at this gestational age (**Figure 2A–C**). The density of NPY immunoreactive fibers was quantified between the fetal CTR, HFD and REV diet groups to determine if differences were present in sympathetic innervation in the fetal liver. We observed no differences in the density of NPY/sympathetic periportal innervation between the CTR, HFD and REV fetal diet groups (χ^2^ = 0.8, 2 d.f., *p* = 0.67, **Figure 2D**).

A double-labeled immunofluorescence study was performed in a subset of fetal samples to qualitatively evaluate the colocalization of NPY with TH in the fetal macaque liver. We observed that NPY is closely associated with TH immunoreactivity in the fetal macaque liver. TH and NPY immunofluoresence are localized to the portal triad with very little penetration into the surrounding lobule (**Figure S3**).

### Sympathetic Innervation in the Juvenile Macaque Liver

TH immunohistochemistry was utilized to evaluate the distribution of sympathetic innervation in one-year old juvenile macaques. In contrast to fetal liver, TH immunoreactivity was observed not only in the portal triad, but robust innervation of the hepatic parenchyma was also found in CTR/CTR liver. We observed that TH positive fibers were distributed throughout the stromal compartment of the triad, with concentrations of fibers localized around the portal vein, hepatic artery and bile duct. From the distribution of fibers in the portal triad, TH positive fibers were observed extending into the hepatic parenchyma (**Figure 3A–B**). A qualitative assessment in the CTR/CTR livers suggested that the density of parenchymal TH fibers were higher in the periportal region and less abundant in the distal lobule. No TH immunoreactivity was observed in association with the central vein (data not shown).

We evaluated the co-localization of TH and NPY immunoreactivity in the juvenile liver by double-labeled immunohistochemistry. As in fetal tissue, we observed clear co-localization of NPY immunoreactive fibers with TH immunoreactive fibers in the portal triad (data not shown). Robust NPY and TH immunofluorescence was also present and clearly co-localized in the hepatic parenchyma of juvenile macaques (**Figure 3C–E**).

### High-Fat Diet and Sympathetic Innervation in the Juvenile Macaque

We performed a quantitative evaluation of the effects that exposure to a HFD *in-utero* and in postnatal life had on hepatic sympathetic innervation in our model. We examined the density of TH immunoreactive fibers between the CTR/CTR and HFD/HFD juvenile diet groups to determine if differences were present in sympathetic innervation in the juvenile liver. We evaluated TH immunoreactivity from both periportal regions and parenchymal regions.

Sexually dimorphic differences in sympathetic innervation between CTR/CTR and HFD/HFD juvenile liver were observed in both periportal and parenchymal hepatic regions. Without regard to the sex of the animal, no differences were observed in either periportal or parenchymal regions between CTR/CTR and HFD/HFD animals (periportal; χ^2^ = 0.25, 1 d.f., *p* = 0.6, parenchyma; χ^2^ = 1.5, 1 d.f., *p* = 0.23, **Figure 4A–B**). When separated by sex, females displayed no significant differences in hepatic innervation in either periportal or parenchymal regions, although a trend for increased innervation was observed in the periportal region (periportal; χ^2^ = 2.9, 1 d.f., *p* = 0.9, parenchyma; χ^2^ = 2.2, 1 d.f., *p* = 0.14, **Figure 4C–D**). Males on the other hand, had significantly lower levels of sympathetic innervation in both periportal and parenchymal regions (periportal; χ^2^ = 4.1, 1 d.f., *p* = 0.04, parenchyma; χ^2^ = 8.1, 1 d.f., *p* = 0.005, **Figure 4E–J**).

To confirm that the differences in hepatic innervation we observed were not a result of liver hypertrophy, we analyzed the wet liver weights of the juvenile animals. We observed no statistical differences in gross liver weight between the CTR/CTR and HFD/HFD diet groups or when separated by gender (CTR/CTR vs. HFD/HFD: mean 65.2 g and 68.9 g respectively; χ^2^ = 0.4, 1 d.f., *p* = 0.54, CTR/CTR vs. HFD/HFD males: mean 64.4 g and 72.6 g respectively; χ^2^ = 1.5, 1 d.f., *p* = 0.22, CTR/CTR vs. HFD/HFD females: mean 66.2 g and 62.6 g respectively; χ^2^ = 0.2, 1 d.f., *p* = 0.65, **Figure S4A**). Further, when liver weights were normalized to total body weight obtained at necropsy, we observed a significant decrease in the proportional weight of the liver in HFD/HFD juvenile offspring (CTR/CTR vs. HFD/HFD: mean 0.027 and 0.025 respectively; χ^2^ = 5.4, 1 d.f., *p* = 0.02). When separated by gender, a significant decrease in the proportional weight of the liver of HFD/HFD females was observed (CTR/CTR vs. HFD/HFD females: mean 0.027 and 0.024 respectively; χ^2^ = 5.0, 1 d.f., *p* = 0.03). No differences in the proportional weight of the liver were observed for HFD/HFD juvenile males (CTR/CTR vs. HFD/HFD males: mean 0.026 and 0.025 respectively; χ^2^ = 1.5, 1 d.f., *p* = 0.22, **Figure S4B**).

### Hepatic Glycogen Content

To begin to evaluate whether changes in liver innervation may have an effect on hepatic glucose homeostasis in our model, we quantified liver glycogen content. We found that without regard to sex, a non-significant increase in glycogen was observed in the HFD/HFD liver (**Figure 5A**). When separated by sex, no significant differences were observed in hepatic glycogen content in female HFD/HFD animals when compared to CTR/CTR (**Figure 5B**). However, a significant increase in hepatic glycogen content was observed in male HFD/HFD animals when compared to CTR/CTR (**Figure 5C**).

## Discussion

The present study focused on the development of hepatic innervation in the context of both normal and maternal HFD exposure in fetal and juvenile macaques. Here we report novel findings that sympathetic innervation is present in the NHP fetal liver and that decreases in sympathetic innervation are observed in juvenile males exposed to a chronic HFD. Further, expression of inflammatory markers and hepatic apoptosis confirmed the persistence of metabolic stress in the juvenile animals, extending findings previously reported in HFD NHP fetuses [11], [12]. It is clear to us that our model is very complex and the results presented in this report encompass a wide range of developmental timepoints and experimental parameters. Thus, we have included a table that visually organizes our significant findings by developmental timepoint and gender across the fetal and juvenile studies described herein (**Table 4**).

### Hepatic Inflammation

It is currently accepted that pathologies related to poor nutrition and obesity are associated with sustained low-grade inflammation in the circulation and in metabolic tissues [31]–[33]. In the HFD/HFD juvenile liver, although we did not define the cell type(s) responsible, we observed this type of chronic inflammation with increased expression of lymphotoxin-α (LTA) and a trend of increased expression of TNF-α, IL-1β and C-reactive protein (CRP).

The combination of subtle elevations in several pro-inflammatory proteins (TNF-α, IL-1β and CRP) in the HFD/HFD diet group may contribute to the development of an insulin resistant phenotype. Selective constitutive activation of NFκB signaling in hepatocytes produced mild elevations of TNF-α and IL-1β in mouse liver and an association with severe hepatic insulin resistance [34]. CRP is an additional NFκB dependent inflammatory mediator that is produced by hepatocytes [35]. Interestingly, hepatic CRP expression was significantly increased only in the female HFD/HFD juveniles in our model. However, a trend for higher expression was observed across both sexes in HFD/HFD animals. The reason for the apparent sexual dimorphism of CRP expression is unclear.

LTA is a soluble factor produced by lymphocytes as well as a component of a membrane heterocomplex with lymphotoxin-β (LTB) [36], [37]. Lymphotoxin signaling has been shown to be involved with lymphoid organogenesis, splenic architecture, natural-killer (NK) and natural killer-T cell (NKT) differentiation, and formation of tertiary lymphoid tissue and inflammation [38]. Having such broad roles in the development of the immune system makes LTA an interesting marker of the long-term effects of chronic HFD exposure.

Importantly, we found that expression of CHRNA7, a critical component of the cholinergic anti-inflammatory pathway, was significantly reduced in both male and female HFD/HFD juvenile liver. As part of the efferent arm of the inflammatory reflex, acetylcholine release in target tissue by the vagus binds CHRNA7. Signal transduction in macrophages involves pleiotropic signaling cascades that regulate the activation of NF-κB through the inhibition of phosphorylation of IκB, activation of the JAK2/STAT3 pathway and increased expression of suppressor of cytokine signaling (SOCS3) [39], [40]. The net result in macrophages is selective inhibition of inflammatory cytokines with preservation of anti-inflammatory cytokine production [41]. While we were unable to conclusively determine the hepatic localization of CHRNA7 in the juvenile liver, we demonstrated through RT-PCR that macaque CHRNA7 is present in the juvenile liver. Our results suggest that in addition to the presence of increased hepatic inflammation, components of the parasympathetic nervous system involved in anti-inflammatory responses are also decreased in the HFD/HFD juvenile liver.

We previously reported significantly increased levels of hepatic apoptosis were observed in the developing fetal liver [11]. Here we report that increased levels of hepatic apoptosis are found in the HFD/HFD juvenile liver. Interestingly, the increases in apoptosis were observed in a sexually dimorphic manner with significant increases observed only in the male liver. Thus, males seem to be the most susceptible to the toxic insults of chronic inflammation. Gender-related differences in response to various types of stress are observed in the livers of rodent and humans [42], [43]. In addition, the prevalence of nonalcoholic steatohepatitis (NASH) is higher in morbidly obese human males [44]. A number of differences, including the levels of circulating sex hormones and hepatic expression of sex hormone receptors, are proposed to mediate gender-specific pathophysiology in the liver [45].

### Hepatic Innervation

Human hepatic innervation in the portal triads begins at about 20 weeks gestation and reaches adult density near term. Parenchymal innervation is observed at very low density in isolated cases at 40 weeks gestation, proceeding to moderate density in the adult [46], [47]. These findings support the idea that development of portal innervation occurs *in-utero*, while parenchymal innervation and consequent maturity of liver innervation occurs after birth in humans [48]. Although the organization of hepatic sympathetic and NPY innervation in portal and parenchymal regions in the adult macaque is similar to that found in adult humans [25], [49], the ontogeny of hepatic fetal and juvenile innervation has never been explored in the macaque.

In this study, we evaluated the distribution of sympathetic nerve fibers in both fetal and juvenile macaques. Previous work in humans reported that NPY containing fibers were not present in fetal liver and were only expressed in isolated cases at term in portal regions. However, in adult human livers, NPY innervation was observed in both parenchymal and portal regions [46], [47]. We observed robust NPY innervation in the fetal macaque liver that was localized to the portal triad. To our knowledge, this is the first demonstration of NPY innervation in the fetal macaque liver. In addition, we observed strong co-localization of NPY with TH and very little penetration of NPY/TH fibers into the hepatic parenchyma in the fetal liver [30], [50].

In the juvenile liver, TH immunoreactivity was present in the portal triads and the hepatic parenchyma as well. In addition, we demonstrated that TH and NPY immunoreactivity are colocalized in portal regions and the hepatic parenchyma in the juvenile macaque. These findings not only confirm hepatic macaque innervation is in parallel with previous studies in humans [46], but also illustrate a window of postnatal development that can be affected by HFD exposure.

Previously published data from this model demonstrated that the HFD is associated with the abnormal development of hypothalamic neuropeptides and the central serotenergic system [15], [51]. Significant decreases in the acetylcholine-induced vasorelaxation response in the abdominal aorta were also reported with this model [52]. Thus, data from this report adds to the growing evidence delineating the vulnerability of the developing nervous system to the effects of chronic HFD exposure. Our comparison of TH nerve fiber density, as a marker of sympathetic innervation, between the CTR/CTR and HFD/HFD juvenile diet groups revealed a significant decrease in sympathetic innervation in the portal and parenchymal regions of male HFD/HFD livers without consequent increases in liver mass. In contrast, a trend for higher sympathetic innervation in both hepatic regions was observed in the female HFD/HFD liver suggesting a gender-specific sensitivity to disruption of hepatic sympathetic innervation in the macaque.

Although we examined the expression of CHRNA7, a parasympathetic receptor involved in an anti-inflammatory pathway, the distribution of hepatic parasympathetic nerve fibers was not addressed in this study.

### Hepatic Function

The functional significance of the sexually dimorphic decrease in hepatic sympathetic innervation we observed has yet to be evaluated in our model. Our findings of increased liver glycogen content in HFD/HFD males and the recent observation that HFD/HFD juvenile males also have significantly increased fasting glucose levels (K.L. Grove, personal communication), suggest that hepatic glucose homeostasis may be affected in a sexually dimorphic manner in our model. Whether the decreases in hepatic sympathetic innervation we observed is playing a role in the increased hepatic glycogen we observed, or hepatic glucose homeostasis as a whole, in our model is currently unknown. Future studies examining hepatic glucose production and hepatic insulin responses will need to be carried out to further define the mechanisms and scope of hepatic pathophysiology present in our model.

This study extends previous work with this model [11], [12], [15], [51], and suggests that chronic exposure to a HFD from conception through one year of age is associated with profound changes in the juvenile liver. Hepatic expression of inflammatory cytokines and increased hepatic apoptosis in the juvenile suggests that severe hepatic pathology is present in the HFD/HFD liver. However, the absence of clear-cut hepatic steatosis in the HFD/HFD juvenile liver precludes a definitive diagnosis of NASH. In addition, significant changes in hepatic sympathetic innervation suggest that hepatic function may be altered in male HFD/HFD offspring, although this remains to be tested. In good agreement with previous work, our findings in this study were also independent of maternal obesity and maternal insulin resistance [12]. Taken together, these findings reinforce the impact that continuous exposure to a high-fat diet has in the development of hepatic pathologies and underscores the important public health implications of a modern diet in the emerging obesity epidemic.

## Supporting Information

Figure S1 **Relative expression of C-Reactive protein in female juvenile liver.** Real-Time PCR was used to assess the expression of CRP in one-year old juvenile liver between CTR/CTR and HFD/HFD diet groups. A significant increase in CRP expression was observed in female liver. CTR/CTR; n = 5, HFD/HFD; n = 4, (* = p<0.05).
(TIF)

Figure S2 **RT-PCR amplification of CHRNA7 from juvenile macaque liver.** Following RT-PCR amplification of CHRNA7 from cDNA produced from two randomly chosen juvenile liver samples, the expected 500 bp bands were observed after gel electrophoresis. The presence of authentic macaque CHRNA7 in juvenile liver was confirmed by sequence analysis of the gel-purified bands.
(TIF)

Figure S3 **Co-localization of TH and NPY immunoreactivity in fetal macaque liver by double-labeled immunohistochemistry.** (A). TH immunoreactivity in fetal liver acquired by excitation of tissue sample with 488 nm laser line. (B). NPY immunoreactivity in fetal liver acquired by excitation of tissue sample with 561 nm laser line. (C). Overlay of A and B provides a representative image of the robust colocalization of TH and NPY immunoreactive nerve fibers observed in the portal triads of fetal macaque liver. Scale bar = 40 µm.
(TIF)

Figure S4 **Analysis of wet and wet liver weights normalized to total body weight of juvenile macaques used in this study between CTR/CTR and HFD/HFD diet groups.** (A). Comparison of wet liver weights between CTR/CTR and HFD/HFD diet groups across all animals and gender. (B). Comparison of wet liver weights normalized to body weight between CTR/CTR and HFD/HFD diet groups across all juvenile animals and gender. * = p<0.05.
(TIF)
