# Supplementary figures and images for: Perinatal Exposure to a High-Fat Diet Is Associated with Reduced Hepatic Sympathetic Innervation in One-Year Old Male Japanese Macaques
Source: PLoS One. 2012 Oct 30;7(10):e48119. doi: 10.1371/journal.pone.0048119 (PMC3484148; doi:10.1371/journal.pone.0048119)

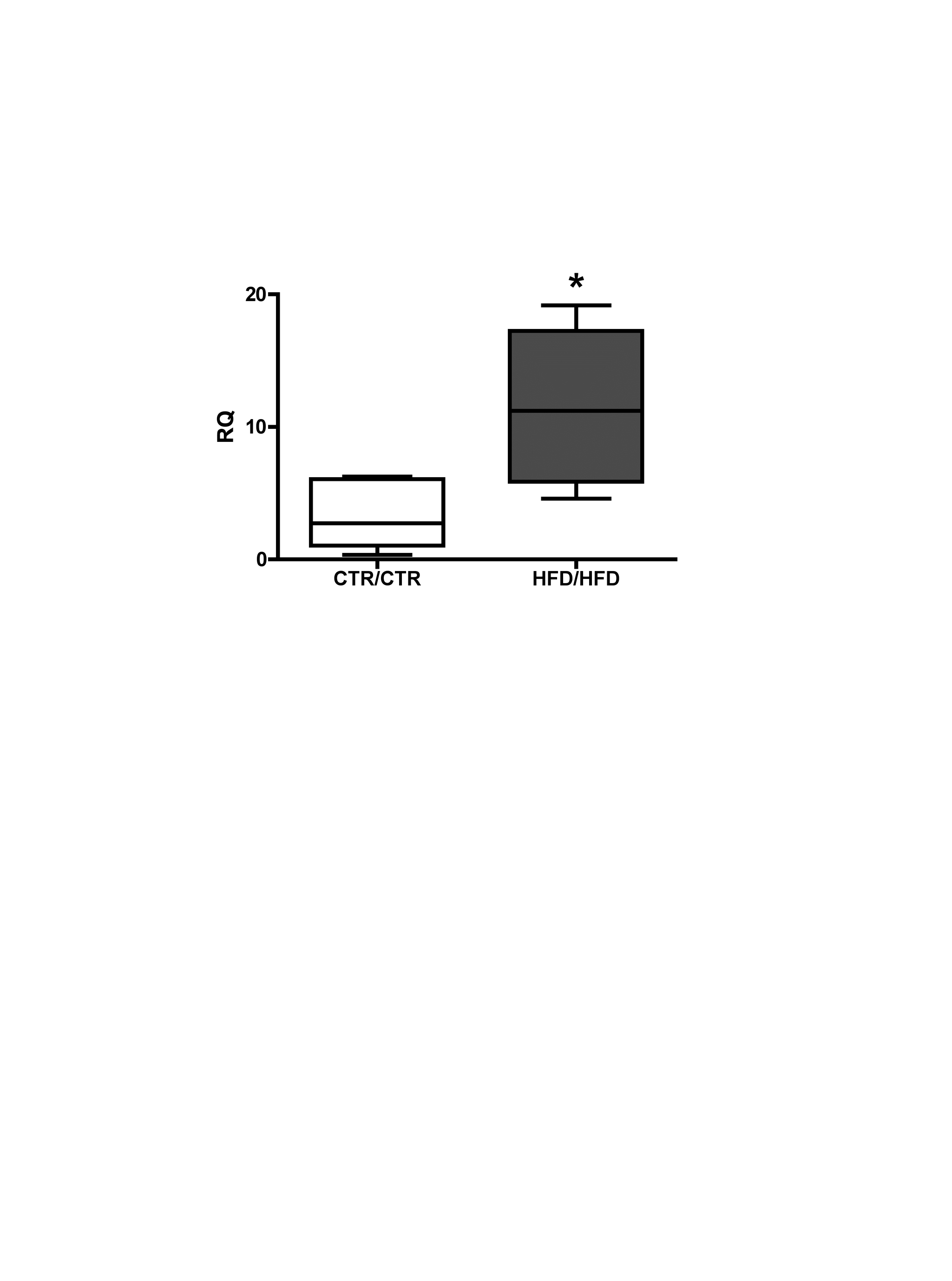

Supplement: Figure S1 — Relative expression of C-Reactive protein in female juvenile liver. Real-Time PCR was used to assess the expression of CRP in one-year old juvenile liver between CTR/CTR and HFD/HFD diet groups. A significant increase in CRP expression was observed in female liver. CTR/CTR; n = 5, HFD/HFD; n = 4, (* = p<0.05). (TIF) [file pone.0048119.s001.tif]

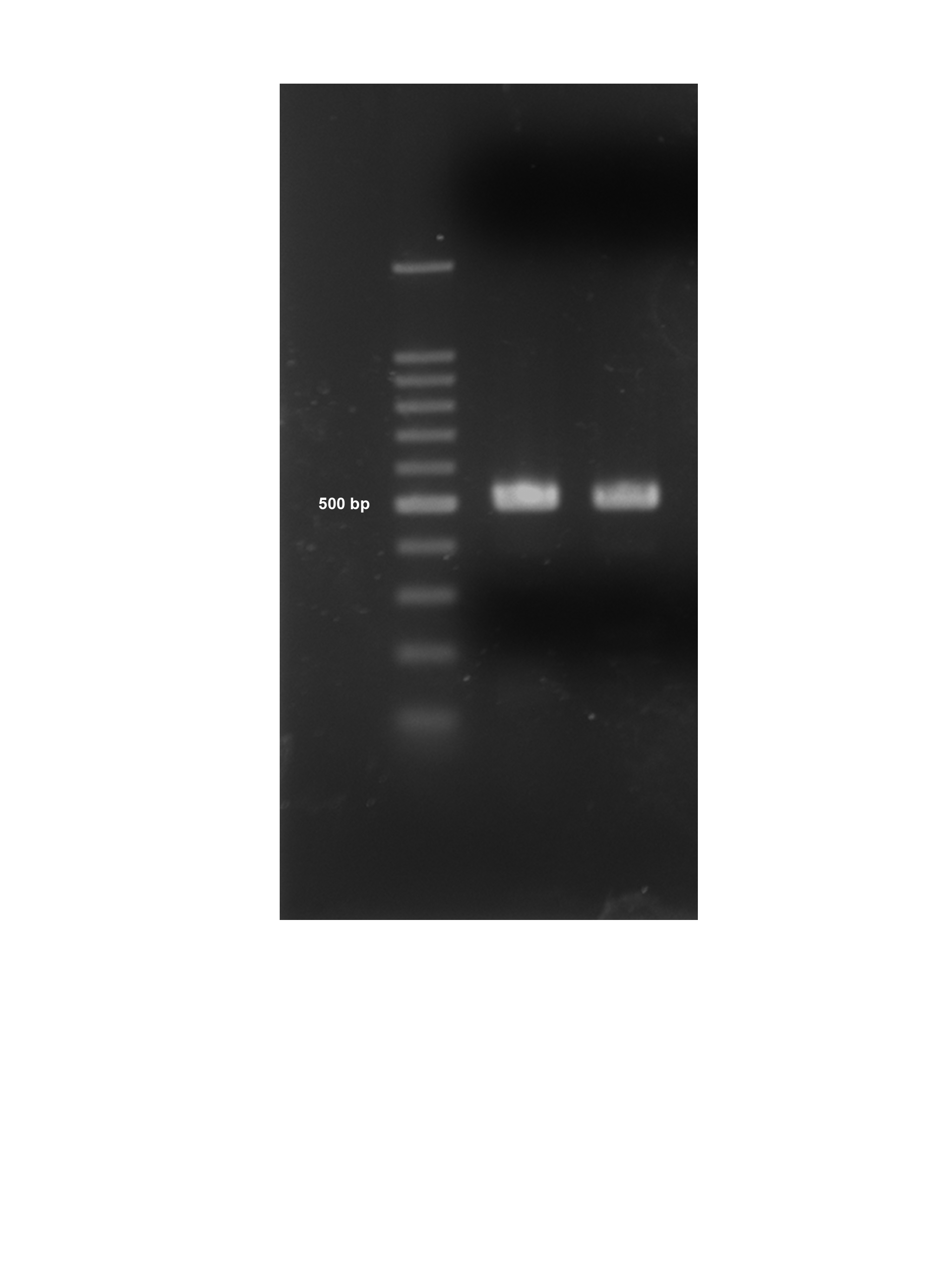

Supplement: Figure S2 — RT-PCR amplification of CHRNA7 from juvenile macaque liver. Following RT-PCR amplification of CHRNA7 from cDNA produced from two randomly chosen juvenile liver samples, the expected 500 bp bands were observed after gel electrophoresis. The presence of authentic macaque CHRNA7 in juvenile liver was confirmed by sequence analysis of the gel-purified bands. (TIF) [file pone.0048119.s002.tif]

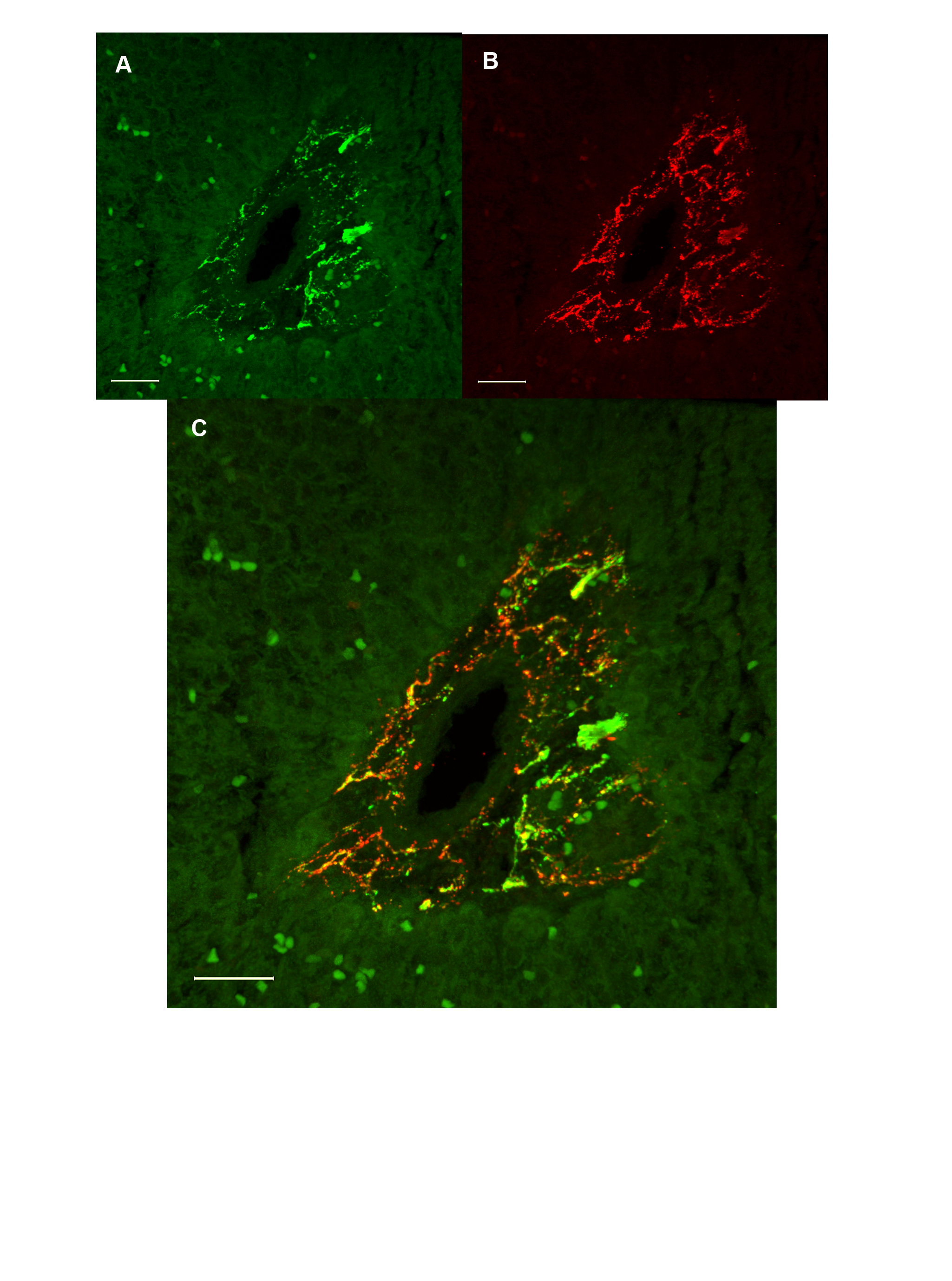

Supplement: Figure S3 — Co-localization of TH and NPY immunoreactivity in fetal macaque liver by double-labeled immunohistochemistry. (A). TH immunoreactivity in fetal liver acquired by excitation of tissue sample with 488 nm laser line. (B). NPY immunoreactivity in fetal liver acquired by excitation of tissue sample with 561 nm laser line. (C). Overlay of A and B provides a representative image of the robust colocalization of TH and NPY immunoreactive nerve fibers observed in the portal triads of fetal macaque liver. Scale bar = 40 µm. (TIF) [file pone.0048119.s003.tif]

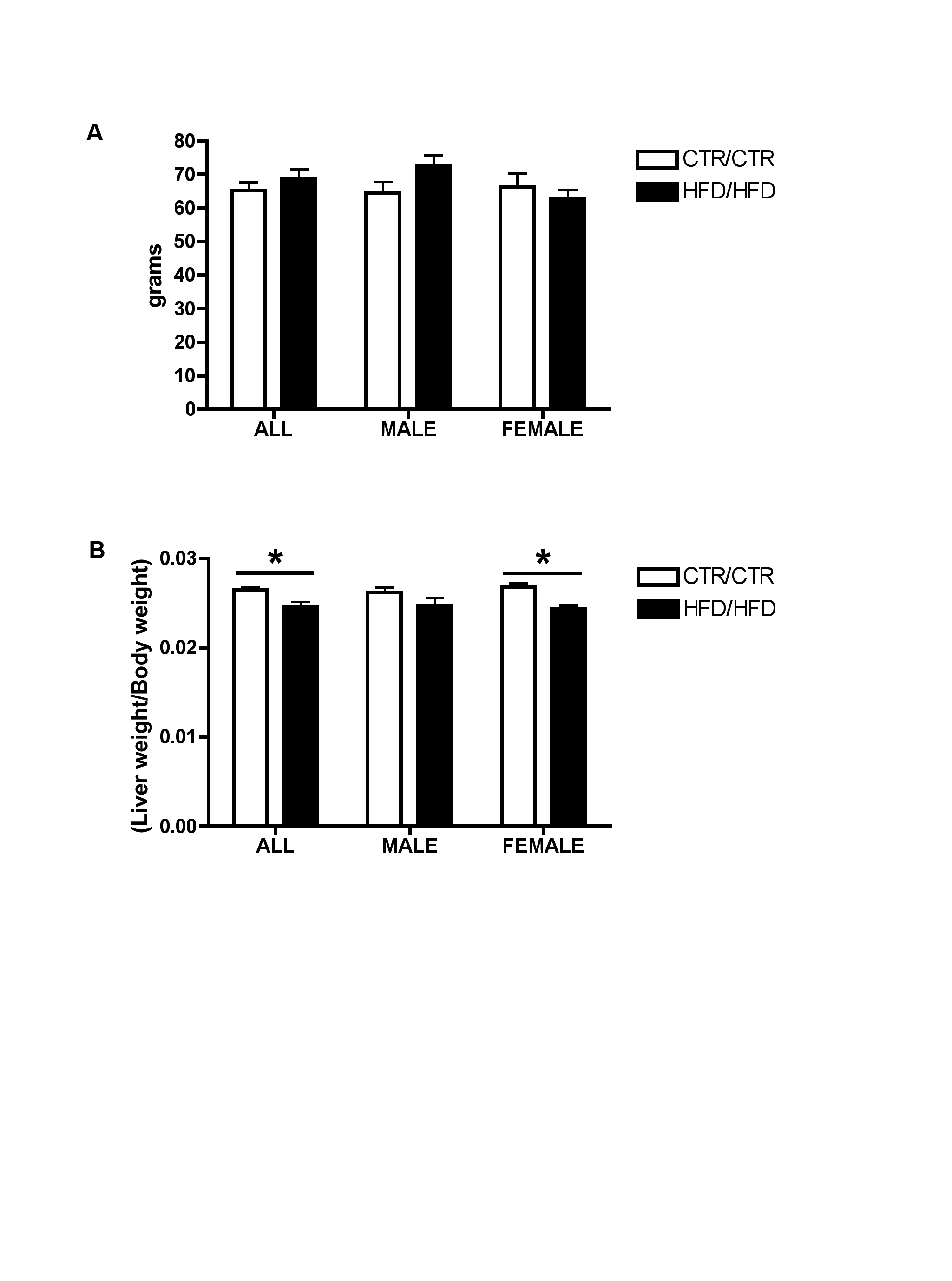

Supplement: Figure S4 — Analysis of wet and wet liver weights normalized to total body weight of juvenile macaques used in this study between CTR/CTR and HFD/HFD diet groups. (A). Comparison of wet liver weights between CTR/CTR and HFD/HFD diet groups across all animals and gender. (B). Comparison of wet liver weights normalized to body weight between CTR/CTR and HFD/HFD diet groups across all juvenile animals and gender. * = p<0.05. (TIF) [file pone.0048119.s004.tif]
